# Parasitism of *Hymenoepimecis manauara* Pádua & Oliveira (Hymenoptera: Ichneumonidae: Pimplinae) on *Leucauge henryi* Mello-Leitão (Araneae: Tetragnathidae) in Brazilian Amazonian

**DOI:** 10.3897/BDJ.4.e11219

**Published:** 2016-12-22

**Authors:** Diego Galvão Pádua, Lidianne Salvatierra, Jober Fernando Sobczak, Marcio Luiz Oliveira

**Affiliations:** 1 Programa de Pós-Graduação em Entomologia, Instituto Nacional de Pesquisas da Amazônia – INPA, Manaus, Brazil; 2 Universidade da Integração Internacional da Lusofonia Afro-Brasileira – UNILAB, Redenção, Brazil

**Keywords:** Cocoon web, ectoparasitoid, parasitoid wasp, *Polysphincta* genus-group, Polysphinctini, spider.

## Abstract

**Background:**

A parasitoid wasp *Hymenoepimecis manauara* Pádua & Oliveira, 2015 was recorded parasitizing, for the first time, a female spider of *Leucauge henryi* Mello-Leitão, 1940 in the Amazon rainforest, Brazil. Images, description of the cocoon and comments about this interaction were added.

**New information:**

First record of *Hymenoepimecis manauara* parasitizing *Leucauge henryi* with description of cocoon and comments about this interaction.

## Introduction

*Hymenoepimecis* Viereck, 1912 belongs to the *Polysphincta* genus-group (Wahl and Gauld 1998), known to be koinobiont ectoparasitoids that attack sub-adults and adults of orb-weaver spiders in the Araneidae, Nephilidae and Tetragnathidae. In some cases, before killing the host spider, the larva induces it to construct a completely modified web structure, called “cocoon web” (e.g. Finke et al. 1990, Eberhard 2000b, Eberhard 2001, Gonzaga and Sobczak 2007, Gonzaga et al. 2010, Sobczak et al. 2009, Sobczak et al. 2014).

The genus is composed by Neotropical parasitoid wasps with occurrence from Mexico to Southern Brazil and also in Cuba (Gauld 1991, Gauld 2000). From the 20 valid species within *Hymenoepimecis*, about ten have well-documented records of their hosts (see Table 1).

*H. manauara* has been recently described based on females and males collected in Brazilian Amazon. This species is characterized by having hyaline wings, hind leg orange, with apex of femur, tibia and tarsus black; metasoma orange with posterior margins of tergites II-IV narrowly black, tergites V+ black; and female with ovipositor 1.1-1.3 times as long as hind tibia (Pádua et al. 2015). Until now, there was no record of host for this species.

*Leucauge* is currently composed by 174 species with a worldwide distribution (World Spider Catalog 2016). In field, they generally spin inclined orb webs, with a varying number of radii and frame lines, and the hub loops are followed by a temporary spiral and the sticky spiral (Eberhard 1987). *L. henryi* can be easily identified by the dorsal abdomen distinguishly colored in silver, two pairs of black stripes on median lateral sides, the lateral abdomen with two pairs of yellow spots, and a shape of epigynum (Mello-Leitão 1940: 26, fig. 4-5).

*Hymenoepimecis* larvae are recorded to parasitize *Leucauge* spiders through at least four different ways, but in all cases the parasitoid larva induces the spider to build a modified web, with a reduction in the number of radial lines and spirals, that Eberhard 2000a named "cocoon web". The first case was reported from Costa Rica by Eberhard 2000a, Eberhard 2000b, Eberhard 2001, who recorded *H. argyraphaga* Gauld, 2000 parasitizing *Leucauge (= Plesiometa) argyra* (Walckenaer, 1841). In this interaction, the modified web is formed by only a few rays and significant reduction in the spiral-orbit part. In the web orbit, the larva constructs a thread to attach the cocoon and keep it suspended in the air. The second case was recorded in São Paulo state, Brazil by Sobczak et al. 2009 and involved *H. japi* Sobczak et al., 2009 parasitizing *L. roseosignata* Mello-Leitão, 1943. In this study, the authors observed that the modified web build by spider also showed a reduction in the number of rays and spirals, the cocoon attached and suspended by a silk thread in the air, being very similar to that observed for Eberhard in the Costa Rica. In these two instances, the modified webs built by the spiders are drastically reduced the only a few reinforced radii and a hub that supports the cocoon of the wasp.

Eberhard 2013 again in Costa Rica, recorded the third case of the parasitism in genus *Leucauge*, leading to manipulation of the web-constructing behavior. He recorded *H. tedfordi* Gauld, 1991 parasitizing *L. mariana* (Taczanowski, 1881), and described that the cocoon web build featured a very reinforced radial lines to provide greater stability to modified web. He also noticed of the hub formed by spirals, going on just a few radial lines where cocoon attached and suspended in the air. He suggested that this modified web is very similar to build by spiders in juvenile stages of development. In the fourth case, Gonzaga et al. 2015 collected adults and sub-adults of *L. volupis* (Keyserling. 1893) parasitized by *H. jordanensis* Loffredo & Penteado-Dias, 2009 in Minas Gerais state. In this interaction they observed that the modified webs featured several reinforced radial lines and the absence of the orbicular part. In addition, the hung by a thread suspended in a three-dimensional wire protection. According to Eberhard 2013 these variations in the form of modified webs are probably a reflection of the different responses of the host to the different substances injected by the larvae of the wasps.

Our study documented the fifth case of behavioral manipulation in *Leucage* spiders, extended the distribution records of *L. henryi*, presented the first record of *H. manauara* parasitizing *L. henryi* and described the host-parasitoid interaction from the Brazilian Amazon.

## Materials and methods

A female spider of *L. henryi* with a larva of *H. manauara* attached to its abdomen was found in July 30, 2016. The parasitized spider was collected and placed into a plastic recipient (23.6 x 22.1 x 9 cm), transported to the insect rearing laboratory at National Institute for Amazonian Research (INPA, Manaus) and reared until the emergence of the adult wasp. Vouchers of the parasitoid and thee spider were deposited in the Invertebrate Collection of INPA.

In the field, digital images of the parasitized spider were taken using a Nikon Coolpix L330. In laboratory, the specimens were examined under a Zeiss Stemi 1000 stereomicroscope. Digital images of adult wasp and cocoon were taken using a DFC420 digital camera attached to a Leica M165C stereomicroscope and combined by using the software Leica Application Suite V3.4.1 (Version 2009). The drawings were vectorized digitally using a vectorization program.

## Taxon treatments

### Hymenoepimecis manauara

Pádua & Oliveira, 2015

urn:lsid:zoobank.org:pub:6857F19D-44C3-4264-9E89-A4232D62D23E

#### Materials

- **Type status:** Other material. **Occurrence:** occurrenceRemarks: Found near Acará Stream, Adolph Ducke Reserve, Manaus, Amazonas; individualCount: 1; sex: female; lifeStage: adult; occurrenceStatus: present; preparations: pinned; disposition: in collection at INPA; occurrenceID: urn:lsid:zoobank.org:pub:6857F19D-44C3-4264-9E89-A4232D62D23E; **Taxon:** taxonID: http://www.gbif.org/species/8477686; scientificName: *Hymenoepimecis manauara* Pádua & Oliveira, 2015; acceptedNameUsage: Hymenoepimecis manauara; parentNameUsage: Ichneumonidae; originalNameUsage: Hymenoepimecis manauara Pádua & Oliveira, 2015; nameAccordingTo: Pádua, Diego G., Oliveira, Marcio L., Onody, Helena C., Sobczak, Jober F., Sääksjärvi, Ilari E., Gómez, Isrrael C. (2015): The Brazilian Amazonian species of *Hymenoepimecis* Viereck, 1912 (Hymenoptera: Ichneumonidae: Pimplinae). Zootaxa 4058 (2): 175-194, DOI: http://dx.doi.org/10.11646/zootaxa.4058.2.2; namePublishedIn: Pádua, Diego G., Oliveira, Marcio L., Onody, Helena C., Sobczak, Jober F., Sääksjärvi, Ilari E., Gómez, Isrrael C. (2015): The Brazilian Amazonian species of Hymenoepimecis Viereck, 1912 (Hymenoptera: Ichneumonidae: Pimplinae). Zootaxa 4058 (2): 175-194, DOI: http://dx.doi.org/10.11646/zootaxa.4058.2.2; higherClassification: Animalia; kingdom: Animalia; phylum: Arthropoda; class: Insecta; order: Hymenoptera; family: Ichneumonidae; genus: Hymenoepimecis; specificEpithet: manauara; taxonRank: species; scientificNameAuthorship: Pádua & Oliveira, 2015; nomenclaturalCode: ICZN; taxonomicStatus: accepted; **Location:** higherGeographyID: TGN: 1022014; higherGeography: Brazil; continent: South America; country: Brazil; countryCode: TGN: 1000047; stateProvince: Amazonas; county: Manaus; locality: Adolph Ducke Reserve; verbatimLocality: Acará stream, Adolph Ducke Reserve; verbatimCoordinates: 2 52 60S 59 58 00W; verbatimCoordinateSystem: degrees decimal minutes; decimalLatitude: -2.1994; decimalLongitude: -57.3806; **Identification:** identifiedBy: D. G. Pádua; dateIdentified: 2016; identificationReferences: Pádua, Diego G., Oliveira, Marcio L., Onody, Helena C., Sobczak, Jober F., Sääksjärvi, Ilari E., Gómez, Isrrael C. (2015): The Brazilian Amazonian species of *Hymenoepimecis* Viereck, 1912 (Hymenoptera: Ichneumonidae: Pimplinae). Zootaxa 4058 (2): 175-194, DOI: http://dx.doi.org/10.11646/zootaxa.4058.2.2

#### Distribution

This species is known from Brazil in Amazonas, Pará and Rondônia states (Pádua et al. 2015).

## Analysis

Our study extended the known distribution of *L. henryi* to Amazonas state. This species has been only previously recorded occurring near Xingu river, Pará state, and there was no information available about its natural history.

**Figure 1.**
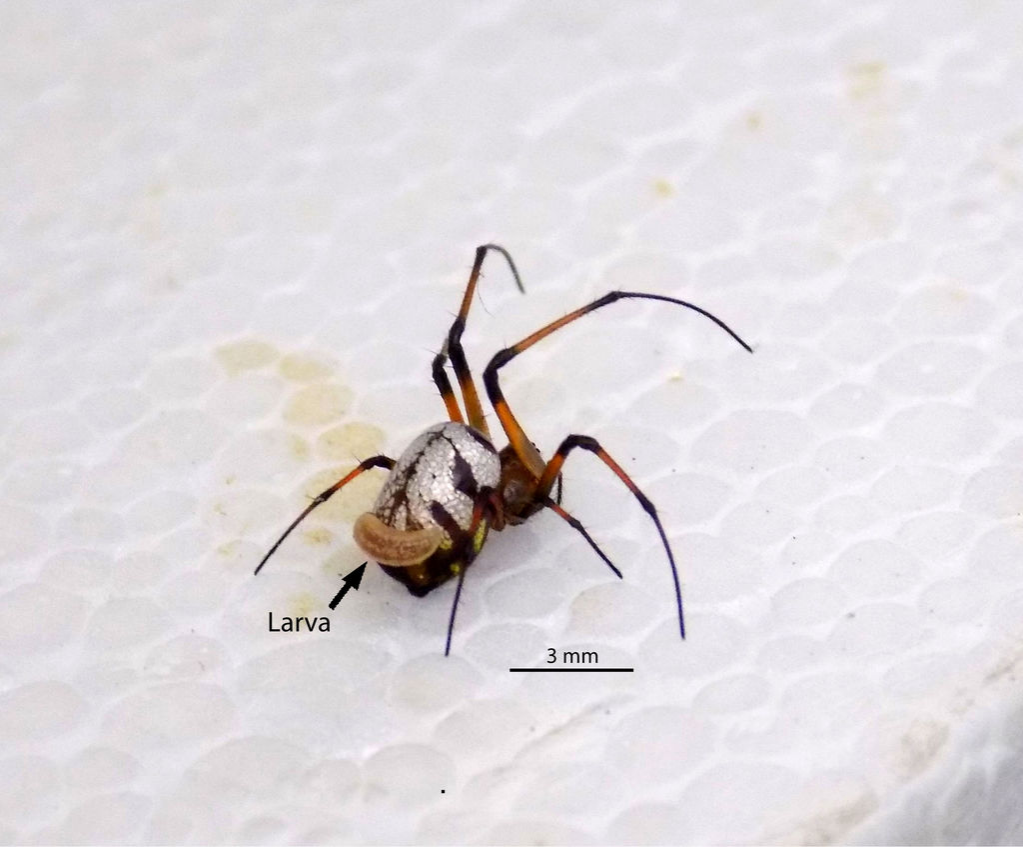
Adult female of *Leucauge henryi* containing a larva of *Hymenoepimecis manauara* attached on its abdomen.

The wasp larva killed the spider two days after it has been capture and a new female of *H. manauara* emerged from the cocoon after 10 days. We observed that the larva remained attached to the postero-dorsal surface of the spider’s abdomen during its development, feeding on its haemolymph (Fig. 1). This same position of parasitoid larvae in abdomen of host spiders had been already observed in *H. bicolor* (Brullé, 1846) on *Nephila clavipes* (Linnaeus, 1767) (Gonzaga et al. 2010), in *H. japi* Sobczak *et al*., 2009 on *L. roseosignata* Mello-Leitão, 1943 (Sobczak et al. 2009), in *H. sooretama* Sobczak et al., 2009 on *Manogea porracea* (C.L. Koch, 1838) (Sobczak et al. 2009), and *H. veranii* Loffredo & Penteado-Dias, 2009 on *Araneus orgaos* Levi, 1991 in South America (Sobczak et al. 2014).

**Figure 2.**
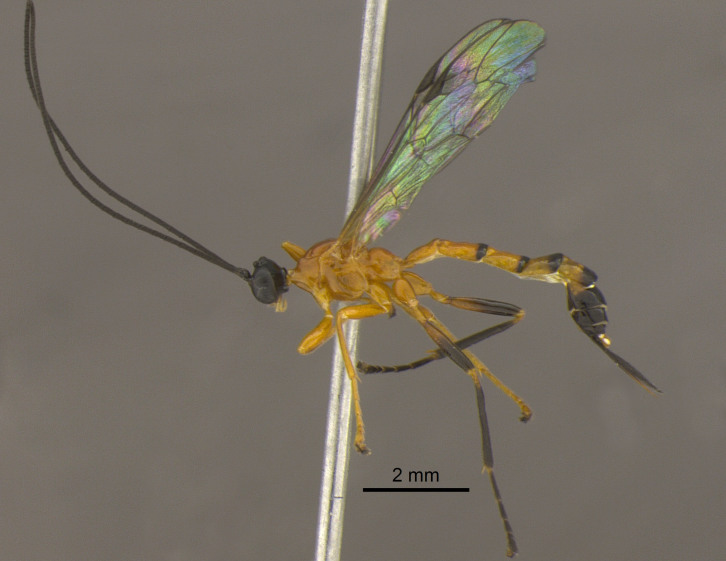
Adult female of *Hymenoepimecis manauara*.

The female and male of *H. manauara* were recently described by Pádua et al. (2015) and were characterized by having the femur orange with black apex. However, the female in our study (Fig. 2) showed an intraspecific variation: femur orange with a half-black apex.

**Figure 3.**
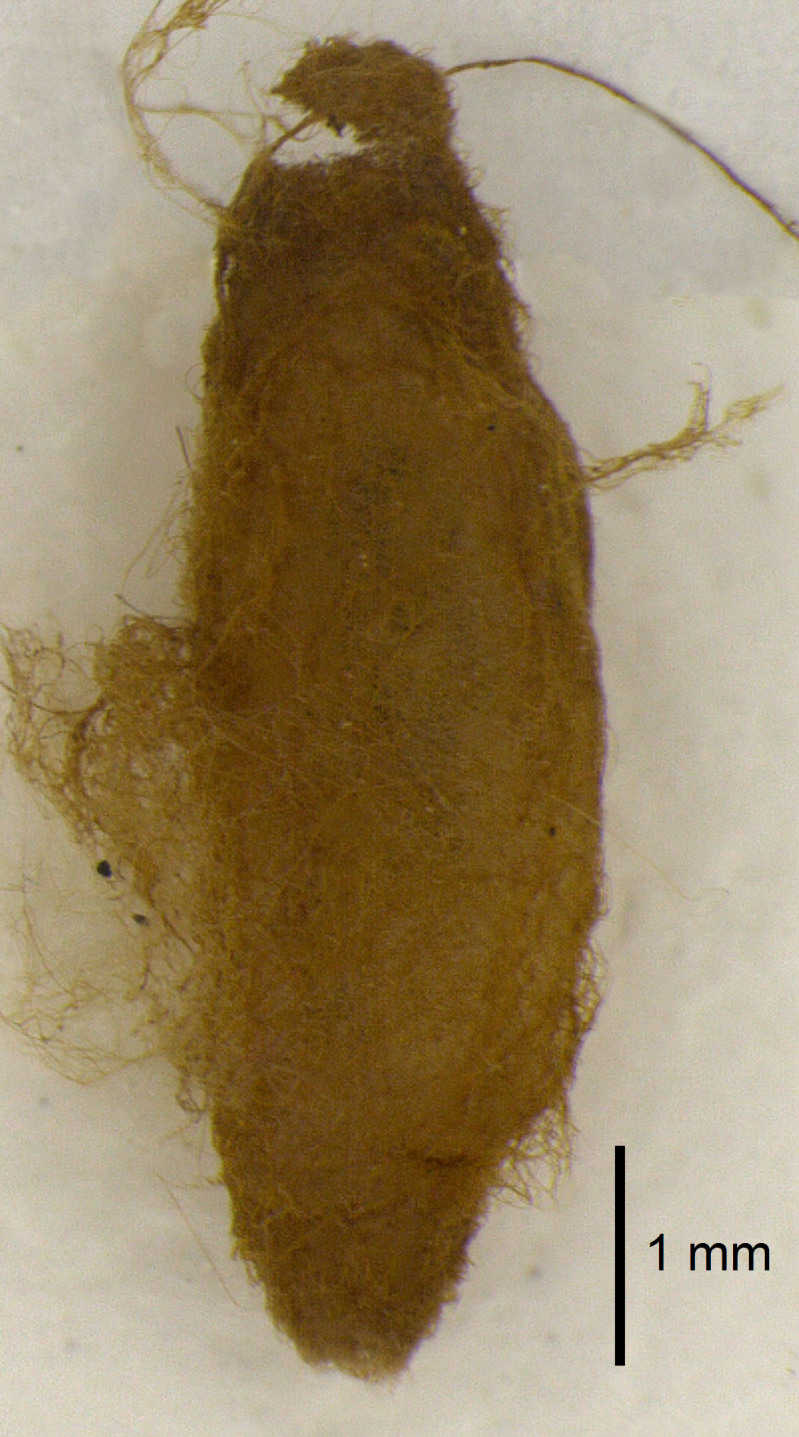
Cocoon of *Hymenoepimecis manauara*.

Cocoon (Fig. 3): Fusiform, without a caudal orifice, about 8.2 mm long and 2.86 mm at its maximum diameter, with silk golden orange color.

## Discussion

**Figure 4.**
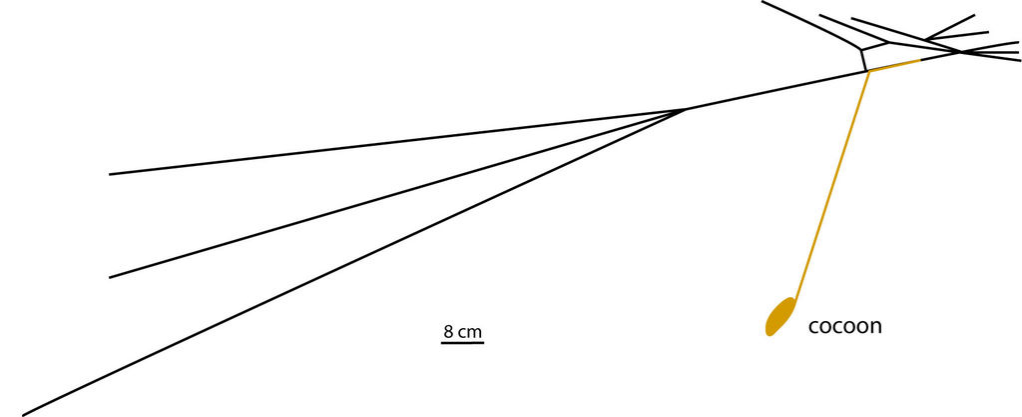
Schematic representation of the cocoon web (black lines = cocoon web produced by the spider *Leucauge henryi*; yellow line = suspension line and cocoon produced by the parasitoid *Hymenoepimecis manauara* larval).

We observed that the parasitoid larva induced a reduction in the number of web threads, in which the larva built the suspension line of the cocoon (Fig. 4). The cocoon web described here was constructed in laboratory, so there is the possibility that space restriction of characteristics of the artificial substrate may influence web patterns.

Similar cocoon webs were observed in the parasitism by *H. argyraphaga* on *L. argyra* (Eberhard 2000a, Eberhard 2000b, Eberhard 2001) and *H. japi* on *L. roseosignatha* (Sobczak et al. 2009). Probably the reduction in the number of radii and sticky spirals determine the reduction in the odds of insect interception, which would result in damages or even in web destruction (Gonzaga et al. 2015). The attachment of the cocoon to this structure may also prevent the cocoon from falling on the ground. Furthermore, this modification makes the web structure more resistant to heavy rains and keeps the pupa wasp from ant attacks, once the cocoon remains suspended on the vegetation.

## Supplementary Material

XML Treatment for Hymenoepimecis manauara

## Figures and Tables

**Table 1.** Summary of the host-parasitoid interactions involving the genus *Hymenoepimecis*.

***Hymenoepimecis* species**	**Host spiders**	**References**
*H. argyraphaga* Gauld, 2000	*Leucauge argyra* (Walckenaer, 1841)	Eberhard 2000a, Eberhard 2001
*H. bicolor* (Brullé, 1846)	*Nephila clavipes* (Linnaeus, 1767)	Gonzaga et al. 2010
*H. heidyae* Gauld, 1991	*Cyrtophora nympha* (Simon, 1895)	Gauld 2000
*H. japi* Sobczak, Loffredo & Penteado-Dias, 2009	*Leucauge roseosignata* Mello-Leitão, 1943	Sobczak et al. 2009
*H. jordanensis* Loffredo & Penteado-Dias, 2009	*Leucauge volupis* (Keyserling, 1893)	Gonzaga et al. 2015
*H. manauara* Pádua & Oliveira, 2015	*Leucauge henryi* Mello-Leitão, 1940	This work (Pádua et al.)
*H. neotropica* (Brues & Richardson, 1913)	*Araneus omnicolor* (Keyserling, 1893)	Sobczak et al. 2012b
*H. robertsae* Gauld, 1991	*Nephila clavipes* (Linnaeus, 1767)	Finke et al. 1990, Gauld 1991, Gauld 2000, Gonzaga et al. 2010
*H. silvanae* Loffredo & Penteado-Dias, 2009	*Araneus venatrix* (Koch, 1838)	Sobczak et al. 2012a
*H. sooretama* Sobczak, Loffredo & Penteado-Dias, 2009	*Manogea porracea* (C.L. Koch, 1838)	Sobczak et al. 2009
*H. tedfordi* Gauld, 1991	*Leucauge mariana* (Taczanowski, 1881)	Gauld 1991, Eberhard 2013
*H. veranii* Loffredo & Penteado-Dias, 2009	*Araneus omnicolor* (Keyserling, 1893), *Araneus orgaos* Levi, 1991	Gonzaga and Sobczak 2007, Sobczak et al. 2011, Sobczak et al. 2014
